# Evaluation of promoting effect of a novel Cu-bearing metal stent on endothelialization process from *in vitro* and *in vivo* studies

**DOI:** 10.1038/s41598-017-17737-9

**Published:** 2017-12-12

**Authors:** Shujing Jin, Xun Qi, Bin Zhang, Ziqing Sun, Bingchun Zhang, Hui Yang, Tongmin Wang, Bo Zheng, Xingang Wang, Qiuping Shi, Ming Chen, Ling Ren, Ke Yang, Hongshan Zhong

**Affiliations:** 1 Institute of Metal Research, Chinese Academy of Sciences, 72 Wenhua Road, Shenyang, 110016 China; 2 School of Materials Science and Engineering, Dalian University of Technology, Dalian, 116024 China; 3 Department of Radiology, The First Affiliated Hospital of China Medical University, Shenyang, 110001 China; 4 Key Laboratory of Diagnostic Imaging and Interventional Radiology of Liaoning Province, The First Affiliated Hospital of China Medical University, Shenyang, 110001 China; 5 Peking University First Hospital, Department of Cardiology, Beijing, 100034 China

## Abstract

Drug eluting stents (DES) have been extensively applied nowadays and reduce the incidence of in-stent restenosis (ISR) greatly as compared with bare metal stents (BMS). However, the development of DES is hindered by the risk of late stent thrombosis (LST) due to delayed re-endothelialization, while endothelialization is an important process related to ISR and LST after implantation. 316L is a traditional stent material without bioactivity and have a high risk of ISR. Cu is recognized for angiogenesis stimulation in these years. Hence a copper bearing 316L stainless steel (316L-Cu) was prepared and evaluated about its effect on endothelialization in this paper. Compared with traditional 316L, it was proved that 316L-Cu increased the proliferation of co-cultured human umbilical vein endothelial cells (HUVECs) at first day. Moreover, HUVECs stretched better on the surface of 316L-Cu. It also improved the expression of angiogenesis related genes and tube formation ability *in vitro*. 316L-Cu-BMS, DES and 316L-BMS were implanted in swine to evaluate the re-endothelialization ability *in vivo*. And 316L-Cu-BMS showed the best effect on endothelialization with good biosafety. Consequently, 316L-Cu is a kind of promising BMS material for coronary field.

## Introduction

The technology of percutaneous coronary intervention (PCI) has been developed in the past decades from percutaneous coronary balloon angioplasty (PTCA) to coronary stent including bare metal stents (BMS) and drug eluting stents (DES)^1–6^. DES, such as polymer-based sirolimus- (Cypher) and paclitaxel-eluting (Taxus) DES, have been extensively applied nowadays and reduced 10% to 20% incidence of in-stent restenosis (ISR) as compared with BMS^3,5^. However, ISR continues to limit the long-term success of the approach of DES. Although DES reduce rates of restenosis, some lesions have emerged as major concerns. Several case reports and observational studies suggested that both Cypher and Taxus DES caused substantial impairment in arterial healing characterized by lack of complete re-endothelialization and persistence of fibrin when compared with BMS. Some studies demonstrated a sustained suppression of neointimal proliferation by DES even 1 year after implantation^1–6^. These lesions would cause the late thrombosis, late restenosis of DES, and thus hampered the development of PCI technology^7–10^.

In the body, trace elements of stable inorganic ions, especially metal ions, act as enzyme co-factors, and stimulate cell signaling pathways towards tissue equilibrium^11–17^. It is well known that copper enhances proliferation of endothelial cells and angiogenesis. Studies have shown that dietary copper supplementation increases myocardial vascular endothelial growth factor (VEGF) levels along with an enhanced angiogenesis^15,18,19^. It is noteworthy that dietary copper restriction causes suppression of VEGF expression in the heart, and copper replenishment recovers myocardial VEGF expression^20^. In addition, copper stimulates VEGF expression in cultured human keratinocytes and promotes angiogenesis and wound healing^18^. Copper ions also have been reported to enhance angiogenesis by stabilizing the expression of hypoxia-inducible factor (HIF-1α), thus mimicking hypoxia, which plays a critical role in the recruitment and differentiation of cells and in blood vessel formation^11^.

In order to reduce the incidence of ISR, a novel coronary stent used material, copper bearing 316L stainless steel (316L-Cu), was designed and manufactured in previous studies, in which appropriate amount of Cu was added in the stainless steel based upon the chemical composition of commercial 316L stainless steel during metallurgical process^21,22^. Thus, 316L-Cu is a kind of integral Cu-containing materials. Nano sized Cu-rich phases would precipitate in the matrix of 316L-Cu and release Cu ions in moist or liquid environment^21,23^. Thus, Cu will exist both surface and internal and we did lots of studies about its observations and analysis of microstructures as depicted in previous studies^24,25^. Meanwhile, most of the properties including roughness and mechanical properties are same to 316L. It is remarkable that 316L-Cu was proved to both reduce the smooth muscle cells proliferation and thrombosis, as well as inhibit inflammation after coronary stent implantation in pre-*in vivo* tests, which is promising for reducing ISR in the future application^22,26^. Mandinov *et al*. also found that ISR could be inhibited directly by oral copper chelation in porcine coronary^27^. Thus, the beneficial influence of Cu ions on reduction of ISR was well recognized in the coronary field. However, based upon the problem of unsatisfactory re-endothelialization or dysfunction of DES, the effect of 316L-Cu on endothelialization or revascularization *in vitro* and *in vivo* should be considered and studied seriously before clinical application.

Thus, the aim of this work is to study the effect of 316L-Cu on endothelialization through *in vitro* and *in vivo* tests including evaluations of the proliferation of human umbilical vein endothelial cells (HUVECs), cell morphology, migration and tube formation ability of cells as well as the expression of some endothelium differentiation genes on the surface of 316L-Cu. Common 316L were served as control group *in vitro* assays. In addition, 316L-Cu stents were implanted in porcine coronary model for 14 days in order to further study their effect on endothelialization. 316L-BMS and DES were served as control groups. Hope that the *in vitro* and *in vivo* tests could provide scientific data for the clinical application of the promising coronary stent material 316L-Cu.

## Results

### Proliferation, vWF synthesis and morphology of HUVECs

**Figure 1:**
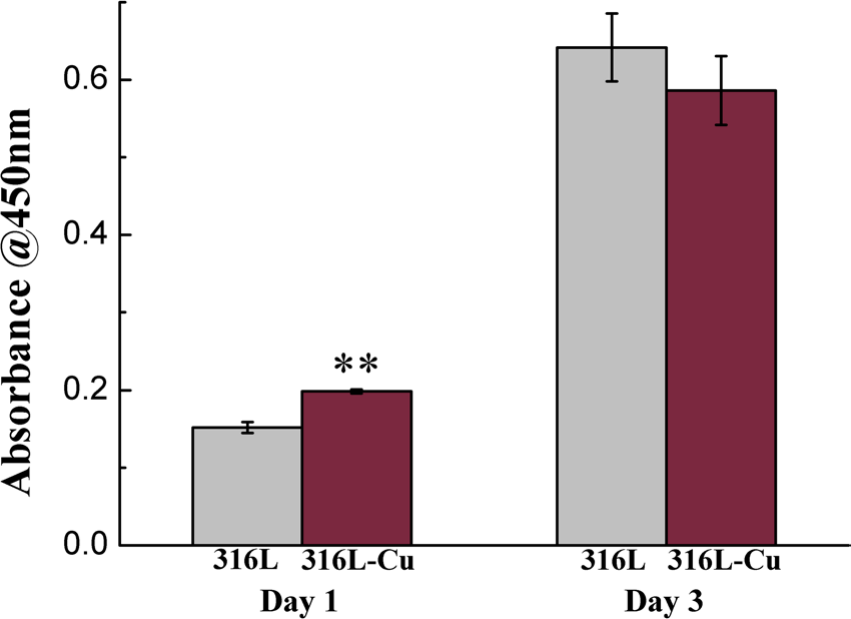
CCK-8 proliferation assay of HUVECs cultured on different substrates for 1 day and 3 days, respectively. **p < 0.01 compared with 316L.

**Figure 2:**
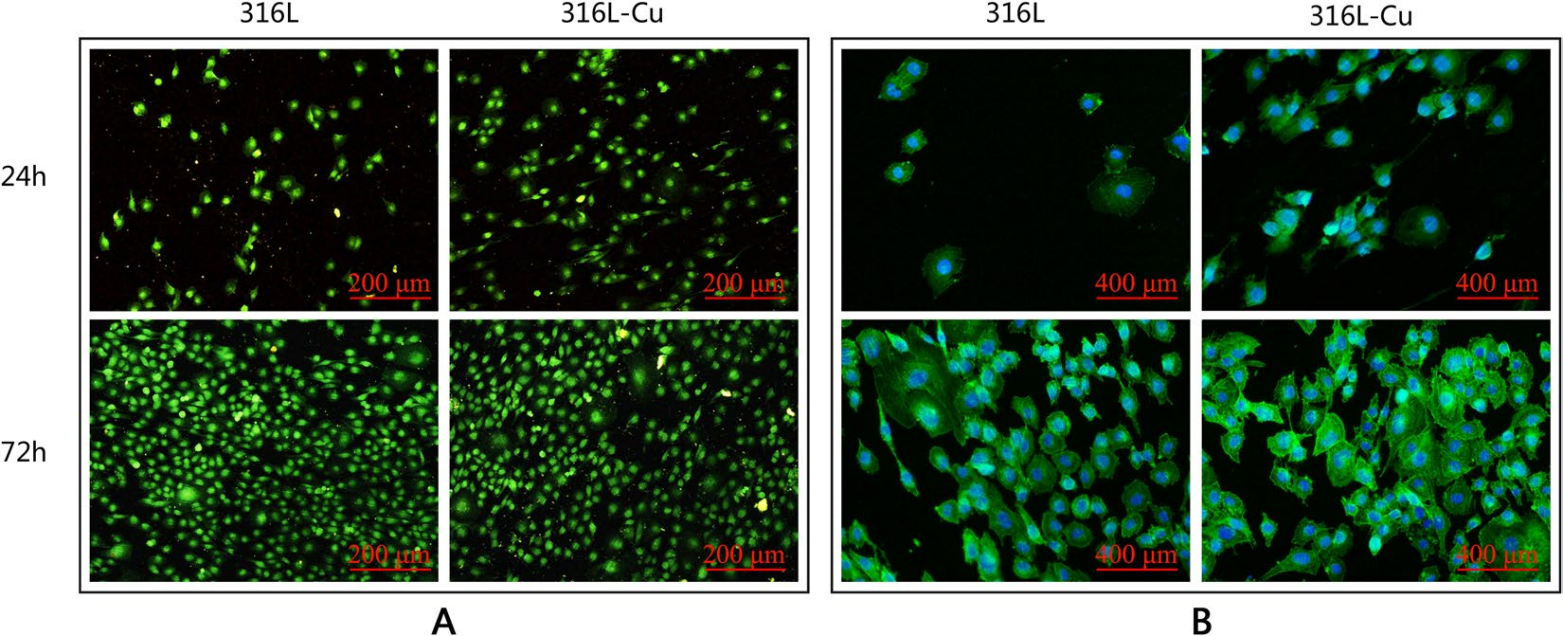
The vWF staining (**A**) and F-actin filament staining (**B**) of HUVECs seeded on 316L and 316L-Cu, respectively.

CCK-8 was performed to compare the proliferation of HUVECs on different substrates for 1 day and 3 days. It could be seen that cells in both groups grew with increasing days (Fig. 1). Compared with 316L, 316L-Cu stimulated cell proliferation at first day significantly and no statistically significant difference existed between them at day 3. In general, 316L-Cu showed no cytotoxicity on HUVECs.

von Willebrand factor (vWF) is a protein synthesized and secreted by endothelial cells and megakaryocytes, which is often used to identify endothelial cells. As shown in Fig. 2A, HUVECs on the surface of 316L and 316L-Cu synthesized vWF normally. Cytoskeleton is an important part for maintaining the cell shape and many physiological activities including attachment, migration, material transport and mitosis. Cytoskeleton staining could show cell morphology on the materials. As shown in Fig. 2B, F-actin filament was stained green, while nuclei was stained blue. Green fluorescence intensity was increasing significantly with culture time, indicating more expression of F-actin. There were larger amount of filopodia extending on 316L-Cu, revealing that 316L-Cu supplied a better surface for HUVECs to survive on.

### Effect of ionic production on the migration and tube formation of HUVECs

**Figure 3:**
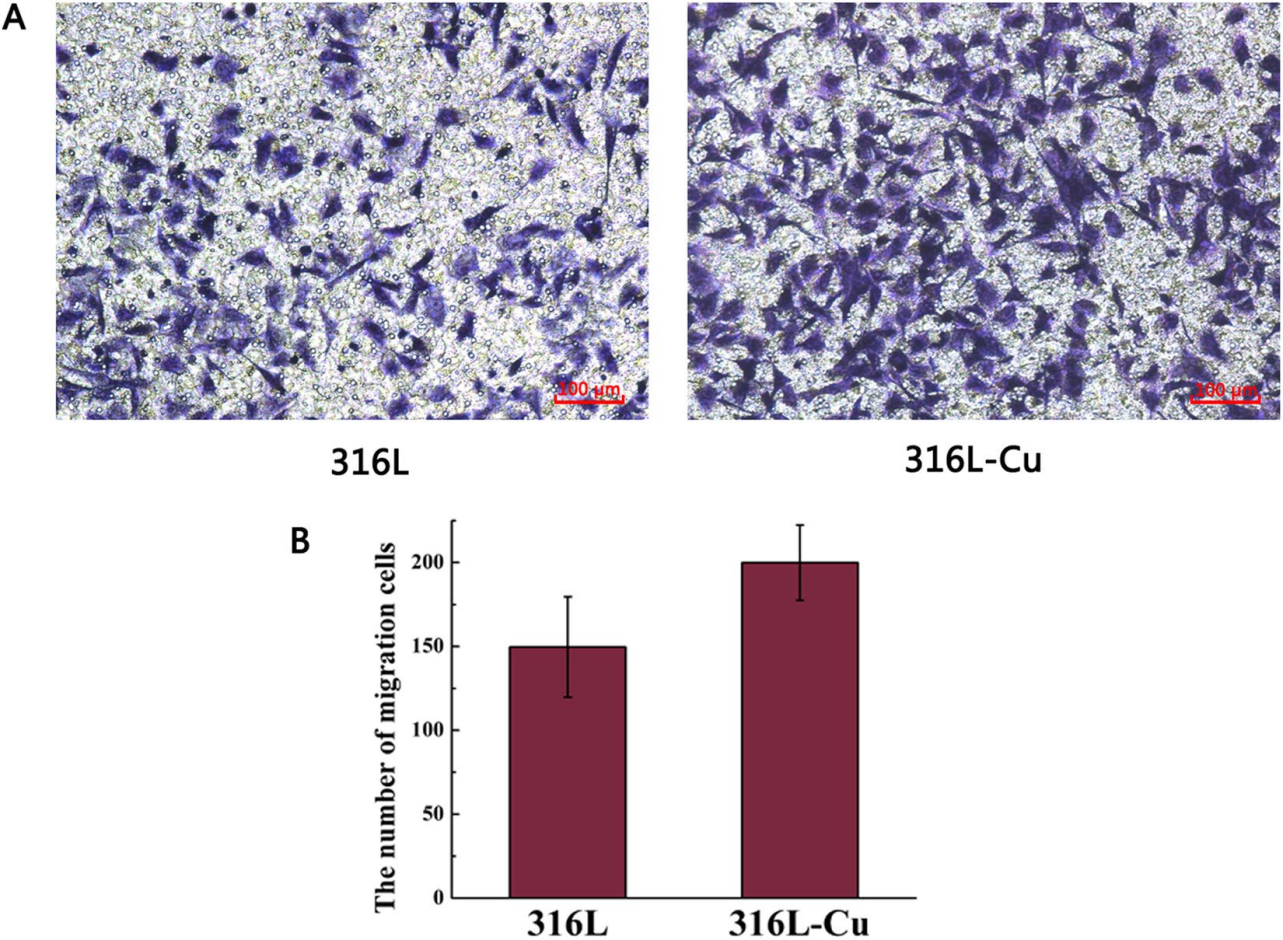
Cell migration induced by the extracts of 316L and 316L-Cu, respectively. HUVECs was stained with 0.1% crystal violet (**A**) and quantified by counting the number (**B**).

**Figure 4:**
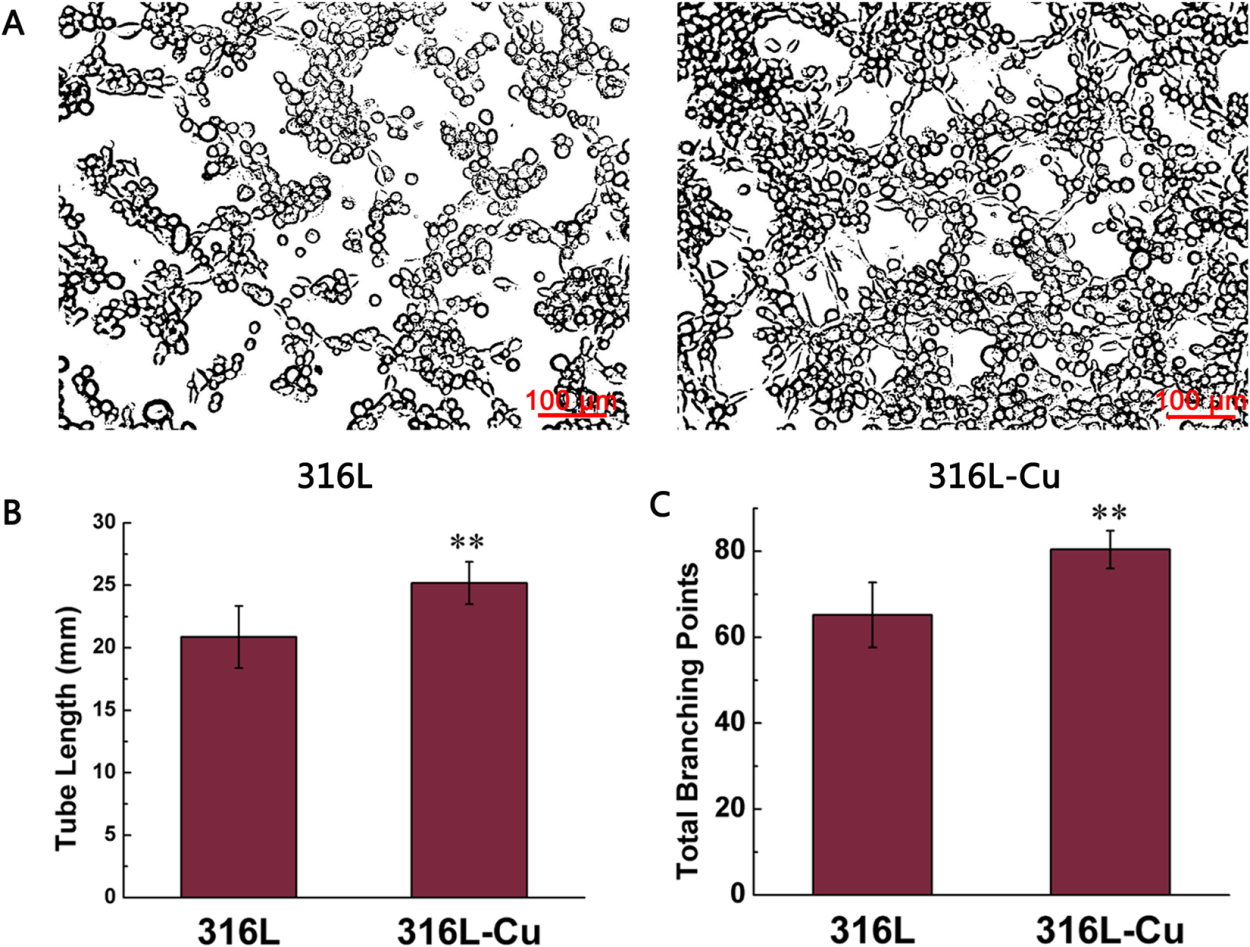
Tube formation induced by the extracts of 316L and 316L-Cu, respectively (**A**). Quantitation of the results by measuring the tube length (**B**) and branching points (**C**). **p < 0.01 compared with 316L.

In transwell migration assay, ionic production attracted cells towards lower compartment. As shown in Fig. 3A, it seemed that more cells moved through the membrane due to the effect of 316L-Cu extracts revealing that 316L-Cu activated cell migration. However, there was no statistically significant difference between the quantitative data of two groups (Fig. 3B). For tube formation assay, HUVECs began to form tube-like structures after incubation on the matrigel. As shown in Fig. 4A, cells cultured with 316L-Cu extracts were connected together to construct network shape structures while tube in control group was not formed almost. The quantitative value of tube length (Fig. 4B) and the number of branching points (Fig. 4C) were higher in 316L-Cu group as compared to 316L group with significant difference. This result indicated that 316L-Cu enhanced migration and tube formation activity of HUVECs in 3D structure.

### The gene expression of VEGF and eNOS

**Figure 5:**
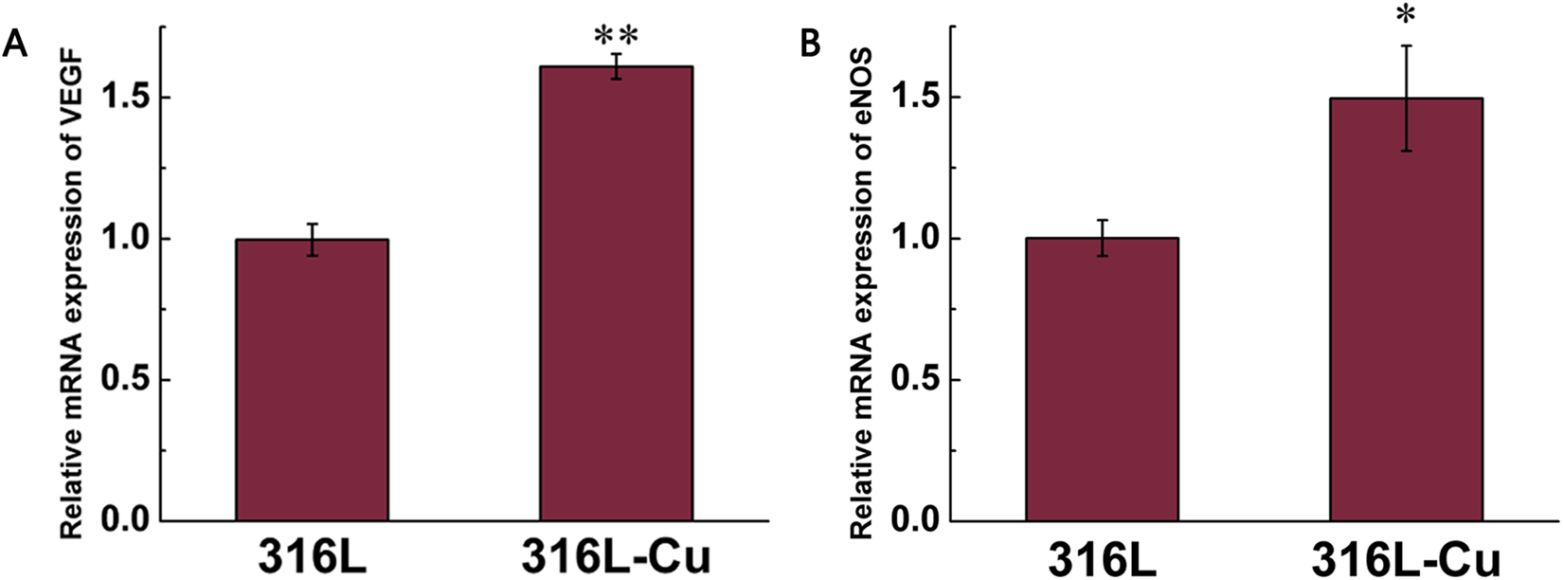
Relative gene expression of VEGF (**A**) and eNOS (**B**) of HUVECs cultured on different substrates for 3 days. *p < 0.05 compared with 316L. **p < 0.01 compared with 316L.

VEGF and endothelial nitric oxide synthase (eNOS) is an important factor and enzyme in angiogenesis, respectively. VEGF is shown to promote the proliferation and migration of endothelial cells. eNOS could synthesize NO that regulates vasodilatation in angiogenesis. After cultured on the surface of 316L-Cu for 3 days, the relative mRNA expression of VEGF was enhanced by 1.6 times (Fig. 5A) while the relative mRNA expression of eNOS was increased by 1.5 times (Fig. 5B). Therefore, 316L-Cu improved the angiogenesis-related gene expression of VEGF and eNOS significantly.

### QCA and OCT results

Based on the above promising *in vitro* results, *in vivo* studies were conducted to evaluate the re-endothelialization influence of 316L-Cu-BMS. A total of 24 stents of three types, including 316L-BMS, DES, 316L-Cu-BMS were successfully implanted in coronary arteries of eight pigs. All of the animals survived in the intended study interval with no angiographic stent thrombosis or other clinical complications. The body weight and body temperature of the animals remained well throughout the study. Quantitative image analysis of coronary angiography (QCA) and optical coherence tomography (OCT) were listed in Tables 1 and 2, respectively. During 14 days, there were no significant differences in all the data statistically among the three groups (P > 0.05), indicating that 316L-Cu-BMS had good biosafety similar to the clinical used 316L-BMS and DES.

**Table 1:** QCA results at 14 day of stent implantation.

QCA results	316L-BMS (n = 8)	DES (n = 8)	316L-Cu-BMS(n = 8)	F	P-value
RVD (mm)	2.51 ± 0.23	2.66 ± 0.32	2.65 ± 0.22	0.760	0.479
DR	1.21 ± 0.12	1.20 ± 0.16	1.09 ± 0.10	2.313	0.123
I-MLD (mm)	2.59 ± 0.25	2.77 ± 0.15	2.75 ± 0.18	1.935	0.168
R-MLD (mm)	2.37 ± 0.20	2.49 ± 0.24	2.47 ± 0.18	0.697	0.509
LL (mm)	0.22 ± 0.35	0.27 ± 0.20	0.28 ± 0.25	0.128	0.880

RVD: reference vessel diameter; DR: dilated ratio; I-MLD: minimal lumen diameter immediately after implantation; R-MLD: minimal lumen diameter of repeat angiography; LL: lumen loss.

**Table 2:** OCT results at 14 day of stent implantation.

OCT results	316L-BMS (n = 8)	DES (n = 8)	316L-Cu-BMS (n = 8)	F	P-value
MSA (mm^2^)	7.06 ± 0.92	7.56 ± 0.54	7.26 ± 0.52	1.191	0.323
MLA (mm^2^)	6.23 ± 0.64	6.70 ± 0.57	6.42 ± 0.49	1.405	0.267
MinSA (mm^2^)	6.48 ± 0.99	6.35 ± 0.92	6.68 ± 0.52	0.374	0.692
MinLA (mm^2^)	5.29 ± 0.79	5.24 ± 0.77	5.55 ± 0.82	0.382	0.687
MinLD (mm)	2.47 ± 0.22	2.49 ± 0.20	2.51 ± 0.19	0.077	0.927
MNA (mm^2^)	0.82 ± 0.43	0.86 ± 0.27	0.84 ± 0.43	0.019	0.981
AS%	11.31 ± 4.59	11.42 ± 3.53	11.53 ± 5.36	0.005	0.995

MSA: mean scaffold area; MLA: mean lumen area; MinSA: minimum scaffold area; MinLA: minimum lumen area; MinLD: minimum lumen diameter; MNA: mean neointimal area; AS: area stenosis.

### SEM evaluation

**Figure 6:**
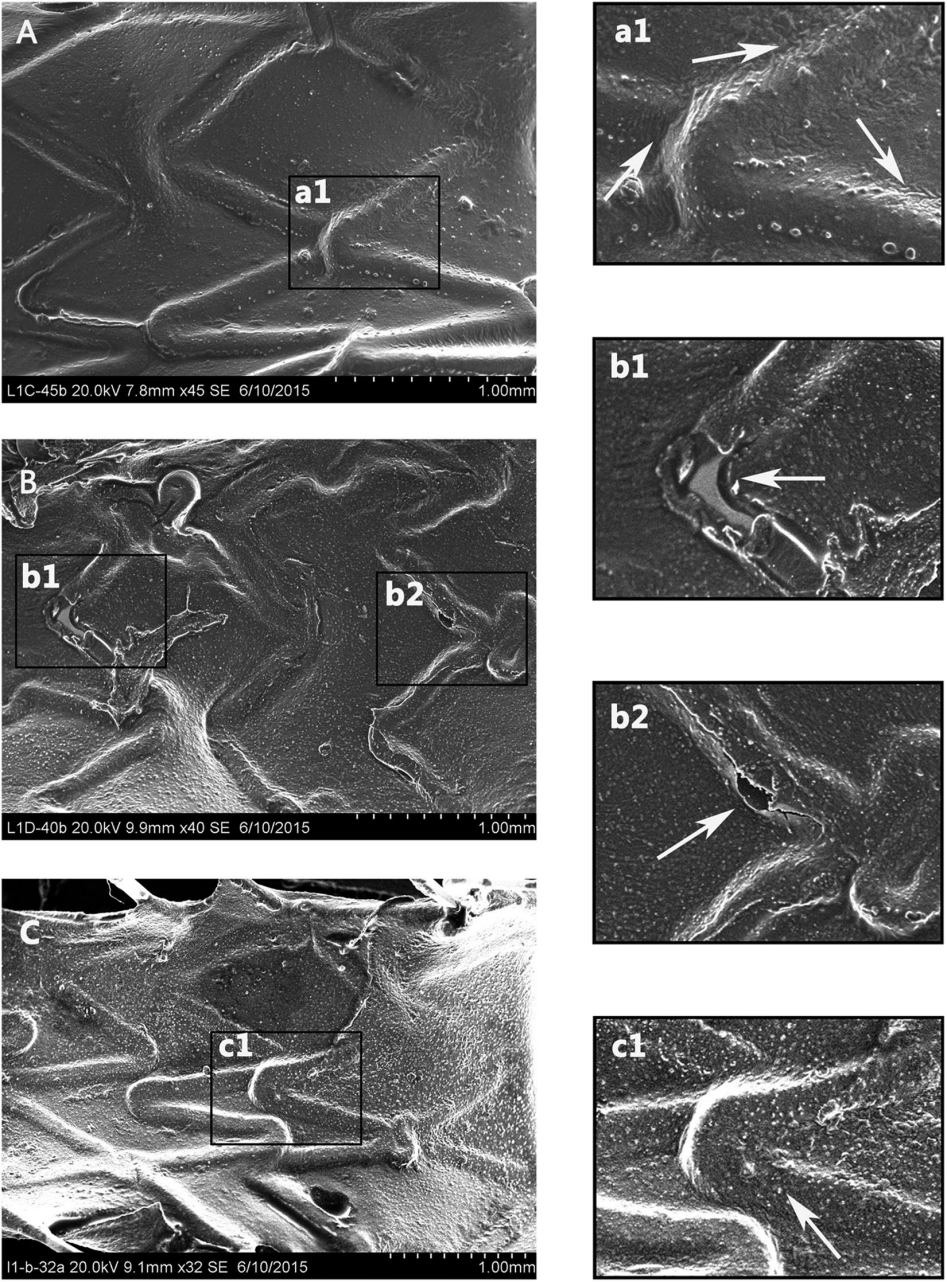
Surface characterization of explanted endovascular stent following 14 days in animal artery. Analysis of endothelial coverage and morphology of endothelial cell layer. Representative scanning electron micrographs: endothelial coverage above struts in 316L-Cu-BMS (**A**), DES (**B**), and 316L-BMS (**C**). a1, b1,b2, c1 are the zoomed-in images indicated by the black squares in A, B, C.

Figure 6 displayed the observed endothelial coverage of the animals’ arteries (more figures were shown in Supplementary Fig. S1). It was reported that plenty of endothelial cells emerged on struts just at 2 days after implant, and its coverage was nearly complete for BMS at 10 days^28^. So there was enough time for the endothelial cell to bind on the surface of stents in this paper. However, re-endothelialization onto the surfaces of struts was varied among the three types stents at 14 days. 316L-Cu-BMS exhibited most complete coverage following interventions in this period (Fig. 6A). The surfaces of the struts were completely covered with regularly shaped endothelia cells in close contact with each other as white arrows in Fig. 6a1, indicating well re-endothelialization. However, incomplete endothelial coverage was observed on stent struts in DES group (Fig. 6B). In Fig. 6b1,b2, the exposed strut was visible, demonstrating poor re-endothelialization ability of DES. Although complete endothelial coverage for 316L-BMS (Fig. 6C), a numerous of particles (white arrow) were observed (Fig. 6c1), which maybe were red cells, white cells and platelets indicating a stronger acute inflammatory and thrombotic response as compared to 316L-Cu-BMS^29^.

### Pathology analysis

**Figure 7:**
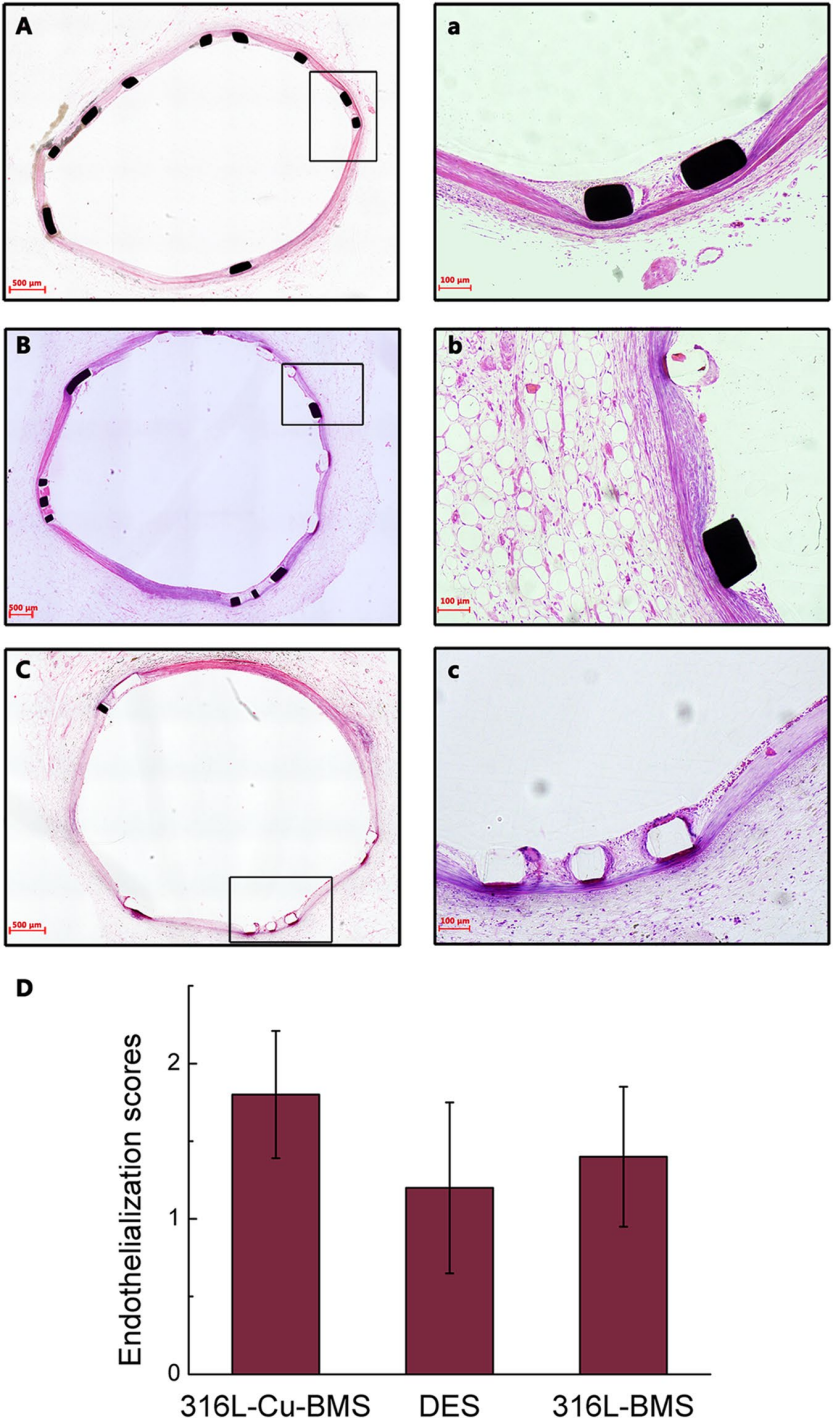
Photomicrographs of representative animal vessel sections after 14 days intervention. Representative Hematoxylin-eosin stain of explanted stents of 316L-Cu-BMS (**A**), DES (**B**), and 316L-BMS (**C**). a, b, c are the zoomed-in images indicated by the black squares in A, B, C, and quantitative morphometric analysis of endothelialization scores (**D**).

In order to further evaluate the effect of re-endothelialization for 316L-Cu-BMS, hematoxylin-eosin (HE) staining was conducted. By day 14, animals were euthanized and the arteries were isolated. Figure 7 showed the typical HE stained images of cross-sections of arteries with stents. Consistent with the result of SEM, 316L-Cu-BMS showed better re-endothelialization as compared to DES. An anatomically intact endothelium had been re-constituted as depicted in Fig. 7A, and the zoomed-in image Fig. 7a reflected that the endothelial just covered on stent struts without neointimal hyperplasia. Whereas, most of the struts of DES were exposed without the coverage of endothelium (Fig. 7B) and the detailed structure could be observed in Fig. 7b, indicating poor endothelialization. For 316L-BMS, the re-endothelialization ability is between 316L-Cu-BMS and DES (Fig. 7C). Some struts are covered by endothelium, and some are exposed (Fig. 7c). Quantitative morphometric analysis of endothelialization scores also revealed that 316L-Cu-BMS showed the best re-endothelialization ability among the three types of stents (Fig. 7D).

## Discussion

DES have reduced rates of restenosis and target lesion revascularization compared with BMS and launched a revolution in the interventional treatment of symptomatic coronary artery disease^1–6^. However, enthusiasm for this technology has been dampened by concerns about delay in arterial healing characterized by persistent fibrin deposition and delayed re-endothelialization, which increase the risk of LST, often with catastrophic consequences^1–10^. T.H. Kim investigated long-term (≥2 years) results of neointimal coverage following sirolimus-eluting stent (SES) and paclitaxel-eluting stent (PES) implantation using OCT. A follow-up angiography with OCT examination was performed in 29 patients with 32 lesions for more than 2 years. They found that incomplete coverage of the stent strut was still observed in a one-fourth of stents and considerable struts were not covered with neointima even 2 years after DES implantation^7^. The majority of DES used in clinical practice are designed to elute pharmacologic agents which could disrupt smooth muscle cell proliferation, such as sirolimus that inhibits the mammalian target of rapamycin (mTOR), a member of the phosphatidylinositol kinase-related family of serine/threonine kinase. However, these inhibitors of mTOR delay the growth and recovery of endothelial cells^1,3,5,6,30^. The first generation of DES, including polymer-based sirolimus and paclitaxel, is modified by lower drug concentrations, which may improve endothelialization and lessen drug toxicity when compared to the prototype. However, the lower drug concentration might enhance the restenosis rate again^1,3,5,30^. Therefore, a simple reduction in drug concentration does not mimic the extended-release stent. Second generation DES such as everolimus eluting stents (EES), Endeavor-zotarolimus eluting stents (E-ZES), and Resolute Integrity zotarolimus eluting stents (R-ZES) are designed with thinner strut backbone stents, less polymer and drug loading, and eluted analogues of sirolimus such as everolimus and zotarolimus, which in some cases have demonstrated superior endothelialization to the 1st generation DES at similar time points^1,3,5,30^. However, delayed re-endothelialization still remains a problem which dampens the development of this technology.

Consequently, the aim of our group is to research and develop a novel kind of BMS without drug coating, which can promote re-endothelialization and then enhance arterial healing process. In our previous studies, we successfully designed and manufactured a novel kind of 316L-Cu, which could be used as BMS material. It is believed that 316L-Cu could release copper ions sustainably and reduce the smooth muscle cells proliferation, thrombosis, inflammation, and promote HUVECs proliferation *in vitro* tests, which is expected to reduce in-stent restenosis *in vivo* ^21,22^. It is commonly accepted that Cu element promotes angiogenesis by stimulation of endothelial cell growth and differentiation. However, it is not clear whether 316L-Cu-BMS could promote endothelialization process caused by Cu ions release from the material. Therefore, the influence of 316L-Cu on endothelialization *in vitro* and *in vivo* was conducted systematically and extensively in the present research in order to provide scientific basement for the clinic application of 316L-Cu that promoting endothelialization and healing process.

The results of CCK-8 and F-actin filament staining assays have demonstrated that 316L-Cu promoted the proliferation and spread of HUVECs. Meanwhile, tube formation assays revealed that 316L-Cu was of great benefit to the migration and tube formation activity of HUVECs. All these above *in vitro* findings indicated that 316L-Cu could enhance endothelialization in contrast to common 316L. It’s no doubt that Cu play a role in promoting migration of endothelial cells. There were lots of studies having the same results^13,17,31^. For example, C. Gerard *et al*. also found that CuSO_4_ significantly induced the formation of tubular structures resembling to an angiogenic process^17^. In summary, Cu release indeed has a great beneficial effect on angiogenesis.

There are several factors involved in the regulation of endothelial cell growth and function, and most studies are about VEGF and eNOS^15,18,19,32^. VEGF has been shown to play a pivotal role in the initiation and formation of blood vessels, which involve endothelial cell growth and differentiation. D.C. Rigiracciolo *et al*. provided novel evidence regarding the molecular mechanisms by which copper may trigger the expression and function of VEGF toward angiogenesis. In particular, they had shown that copper activated the EGFR/ERK/c-fos transduction pathway leading to the expression of HIF-1α, G protein-coupled estrogen receptor (GPER) and VEGF^33^. Ohnishi *et al*. reported that Cu^2+^ elevated the NO level in rat pulmonary arterial rings not by prolonging the half life of NO, but by activating eNOS^34^. However, the mechanism of eNOS activation by Cu^2+^ in pulmonary arterial endothelial cells proposed by Ohnishi *et al*. needs to be explored. Taken together, these results suggest three possibilities: first, Cu^2+^ may activate vascular endothelial NO synthase. Second, Cu^2+^ may prolong the half-life of NO by mechanisms that mimic the action of superoxide dismutase, ultimately resulting in the potentiation of NO activity in vascular tissue. Thirdly, Cu^2+^ stimulates NO release from endogenous *S*-nitrosothiols as Gordge *et al*. described^35^. And S. Li *et al*. demonstrated that Cu at physiologically relevant levels stimulated growth of HUVECs in culture which was eNOS dependent^19^. Consequently, the effect of Cu^2+^ on endothelialization is well recognized and explained. In this present work, 316L-Cu also improved expression levels of angiogenesis-related genes of VEGF and eNOS significantly in HUVECs, indicating that Cu^2+^ release from the steel material could also play the role of endothelialization. Li *et al*. also found that the released Cu^2+^ ions from a copper containing bioactive glass nanocoating on natural eggshell membrane successfully stimulated angiogenesis of endothelial cells by the improved expression levels of VEGF, HIF-1α, VEGF receptor 2 and eNOS as well as increased VEGF and HIF-1α protein secretion^15^. All these results well explain why Cu-bearing biomaterials with Cu^2+^ release have the effect of promoting endothelialization^15^.

Based on the above promising results, *in vivo* studies were conducted to further evaluate the re-endothelialization ability of 316L-Cu. For QCA and OCT, there was no significant difference in all the related data and thus it could not indicate the reduction role of 316L-Cu on ISR with these current *in vivo* tests. It is because that we just focused on the evaluation of promoting re-endothelialization of 316L-Cu rather than on ISR in this study, and we did not establish an animal model for ISR. For this reason, no neointimal hyperplasia could be observed for the three types of stents. Although we could not get information about the effect of 316L-Cu on ISR reduction in this study, our previous *in vitro* tests have shown that it can reduce the smooth muscle cells proliferation and migration, thrombosis, and even inflammation and hence it was suggested to reduce ISR^22,26^. It is notable that no significant difference of QCA and OCT data proved that this 316L-Cu-BMS was biosafety, which was equal to the other two clinical application stents.

Further, SEM observation and HE evaluation were performed to measure the re-endothelialization ability of 316L-Cu-BMS. 316L-Cu-BMS showed the best re-endothelialization ability, whereas DES was the worst due to the drug coating, and 316L-BMS was between the first two. Consequently, 316L-Cu-BMS without drug coating could not only reduce ISR but also promote re-endothelialization. Thus, it is possible that patient may reduce the medicine intake for inhibiting thrombosis, such as clopidogrel, and thus decrease the side effects of medicine to human body, if 316L-Cu-BMS could get clinical application.

Recently, researches regarding to Cu-bearing materials, being as a novel implant material with numerous bio-functions including antimicrobial activity, promoting osteogenesis and even angiogenesis due to the release of Cu ions, are springing up vigorously^21,26,36–42^. However, some researchers especially doctors worried about the biocompatibility of Cu-bearing materials because that Cu is a kind of heavy metallic element. It is true that the biocompatibility of novel biomaterial should be seriously considered before clinical application. For this reason, a large number of evaluations of *in vitro* and *in vivo* tests have done for this Cu-bearing stainless steel in our previous work and parts of the present work. The amount of Cu ions released from 316L-Cu has been measured before, approximately 0.0076 μg (0.12 μM)/day/cm^2^, which is much lower than the cytotoxicity limit of Cu against vascular endothelium cells of 500 μM^21^. For another instance, the approximately surface area of a 316L-Cu-BMS is around 0.34 cm^2^. According to this, a 316L-Cu-BMS stent would release 0.0026 μg Cu^2+^, which is much lower than the acceptable daily intake of Cu (2–3 mg per day per person) recommended by the World Health Organization^21^. Thus, from the above data analysis, 316L-Cu is biosafety for human body. Although the release amount of Cu is trace grade (ppb), what we measured is just the concentration of “planktonic” Cu ions in extracts. The concentration of Cu ions “trapped” on the surface is much higher than that. Others also found the same that the ion concentration was higher significantly in the near implant vicinity, which was decreased quickly along the distance away from the implanted alloys^43,44^. For this reason, 316L-Cu-BMS could play promoting effect on endothelialization *in situ*. It means that only when tissue contact 316L-Cu closely, it can play the role directly.

All of the above results indicated that the 316L-Cu exhibited satisfactory effect on re-endothelialization and good biocompatibility both from *in vitro* and *in vivo* tests. However, there are remaining some limitations in this present study, which should be seriously considered in the future research work for the final application in clinical. The promoting re-endothelialization of 316L-Cu for longer implantation (such as 1, 6, and 12 months) and more comprehensive data analysis should be seriously evaluated. Meanwhile in the future animal test evaluation of reduction role on ISR of 316L-Cu should be focused together. Metabolism of copper from 316L-Cu in human body was still unclear. All these should be assessed in the following step.

## Methods

### Preparation of copper-bearing 316L stainless steels

Based on the composition of commercial 316L, 316L-Cu was designed and fabricated by the vacuum induction melting furnace, whose chemical composition was analyzed as (wt.%): Cu 3.77, Cr 17.29, Mo 3.21, Ni 14.63, and Fe in balance. The cast ingot was reheated to 1100 °C, held for 1 h and then hot forged into plates. The forged plates were solution treated at 1000 °C for 0.5 h followed by a water quench, and then aged at 700 °C for 6 h. Commercial 316L was served as control group, and its composition was (wt.%): Cr 18.49, Mo 3.26, Ni 14.16, and Fe in balance. These two kinds of samples were machined into disks of 10 mm and 5 mm in diameters and 1 mm in thickness, respectively. They were mechanically grinded with silicon carbide papers down to 2000 grade and ultrasonically washed in acetone and ethanol. All the samples were sterilized prior to experiments.

### Preparation of the extracts

Samples (Φ10 mm × 1 mm) were soaked in serum free RPMI.1640 medium at the ratio of 1.25 cm^2^/mL, according to the International Standard Organization (ISO 10993-5). They were stored at 37 °C for 72 h. The culture media incubated with the steel samples were collected as extracts.

### Cell culture

HUVECs (KeyGen, China) were cultured in RPMI.1640 medium (KeyGen BioTECH, China) supplemented with 10% Fetal Bovine Serum (Gibco, Life technologies, US), 80 U/ml penicillin, and 0.08 mg/ml streptomycin as recommended by the supplier. They were incubated at 37 °C in humidified atmosphere containing 5% CO_2_ and 95% air. After reaching 80% confluence, cells were passaged by 0.25 wt.% trypsin-EDTA solution (Gibco, US). Cells at passages 3 to 10 were used for subsequent experiments.

### Cell proliferation

The proliferation of HUVECs on different substrates was examined with Cell Counting Kit 8 (CCK-8, Dojindo). The smaller disks were put into 96-well plates firstly. A suspension consisting of 3 × 10^3^ cells was seeded onto the samples. After co-culture for 1 day or 3 days, the solution was replaced by 110 μL mixture of fresh medium (100 μL) and CCK-8 solution (10 μL), followed by an incubation at 37 °C for 1.5 h. Then 100 μL liquid from each well were transferred to a fresh plate. The absorbance at λ = 450 nm was measured by a microplate reader.

### Immunofluorescence staining

Immunofluorescence is a method to observe the vWF and F-acting expression of cells on the surface of different substrates. The samples of 10 mm in diameter were placed in 24-well plates. 100 μL cell suspension at a appropriate density was dropped on each disk. 1 mL culture medium supplemented with 10% FBS was added after cells were adherent. After incubation for 1 day or 3 days, the substrates were washed three times with PBS, fixed in 4% paraformaldehyde for 15 min, and then permeabilized with 0.5% (v/v) Triton X-100 for 10 min. Next was staining process for vWF and F-actin, respectively.

For vWF staining, cells were incubated with a rabbit anti-vWF primary antibody (1:100 dilution) overnight after blocking by 10% goat serum at room temperature for 30 min. Washed the samples and added FITC-labeled goat anti-rabbit second antibody (1:25 dilution) next day. After incubation for 1 h, the vWF expression was visualized using a fluorescent microscope.

For F-actin staining, HUVECs were stained with phalloidin-FITC (Sigma, USA) at a concentration of 5 ug/ml at 37 °C for 1 h. Cells were further stained with DAPI (Beyotime) to visualize the nuclei. An inverted fluorescence microscope was used to observe the results.

### Transwell migration assay

The effect of different ionic extracts on cell migration was evaluated by transwell assay. HUVECs were suspended at a density of 2 × 10^5^ cells/mL with serum free culture medium after 24 h starvation. 100 μL cell suspension was added in the upper chamber with a 8 μm pore. The lower chamber was filled with 600 μL extract supplemented with 5% serum. Washed the inserts with PBS after 24 h and removed the cells in the upper chamber by cotton swabs gently. Then the cells in the underside surface of the upper chamber were fixed with 95% alcohol, and stained with 0.1% crystal violet for dyeing. Finally, select five views randomly under the microscope and calculate the average cell number to represent the migration cell number.

### Endothelial cell tube formation assay

The tube formation activity of HUVECs cultured with different extracts was investigated by observing capillary-like structures in matrigel. 50 μL matrigel (Corning) was transferred to the well in a 96-well plate by cold pipette tips. The plate was placed at 37 °C for 0.5 h to make matrigel solidified. 100 μL cell suspension with 5 × 10^4^ cells were seeded per well and 100 μL ionic dissolution product with 10% serum was added. After incubation at 37 °C for 2 h, the tube formation of different groups was observed under a light microscope. Quantitative data including tube length and branching points were analyzed by WimasisWim Tube.

### Real-time quantitative PCR (RT-qPCR)

Gene expression of VEGF and eNOS from cells cultured on different substrates was detected by RT-qPCR. The larger disks were placed in 48-well plates, and 3 × 10^4^ cells were seeded per well. Three days later, total RNA of cells was extracted using a Trizol reagent (Vazyme) according to the manual. Cells in every three wells were collected as one sample. Three samples were performed for each group in each independent experiments. The RNA concentrations were measured on a nanodrop 1000 spectrophotometer (Thermo Scientific). About 400 ng of the extracted total RNA was reversely transcribed for cDNA with TaKaRa PrimeScript^TM^RT reagent Kit (Perfect Real Time) in 10 μL system. The following primers were designed by the information in NCBI database and the specificity was confirmed by Primer-BLAST: VEGF, forward 5′-AGTCCAACATCACCATGCAG-3′ and reverse 5′-TTCCCTTTCCTCGAACTGATTT-3′; eNOS, forward 5′-CATCTTCAGCCCCAAACGGA-3′ and reverse 5′-AGCGGATTGTAGCCTGGAAC-3′; GAPDH, forward 5′-GGAGCGAGATCCCTCCAAAAT-3′ and reverse 5′-GGCTGTTGTCATACTTCTCATGG-3′. 2 μL of cDNA, which was diluted with 5 μL RNase free dH_2_O, was mixed with 10 μL SYBR Premix, 6.4 μL dH_2_O, and 1.6 μL primers at the concentration of 10 μM to prepare reaction solution according to TaKaRa SYBR^®^ Premix Ex Taq^TM^ II (TliRnaseH Plus). Real-time PCR assays were performed on the LightCycler480. The mean cycle threshold (Ct) value of each target gene was normalized against Ct value of GAPDH and the relative expression was analyzed using the 2^−ΔΔCT^ method.

### Stents manufacture

**Figure 8:**
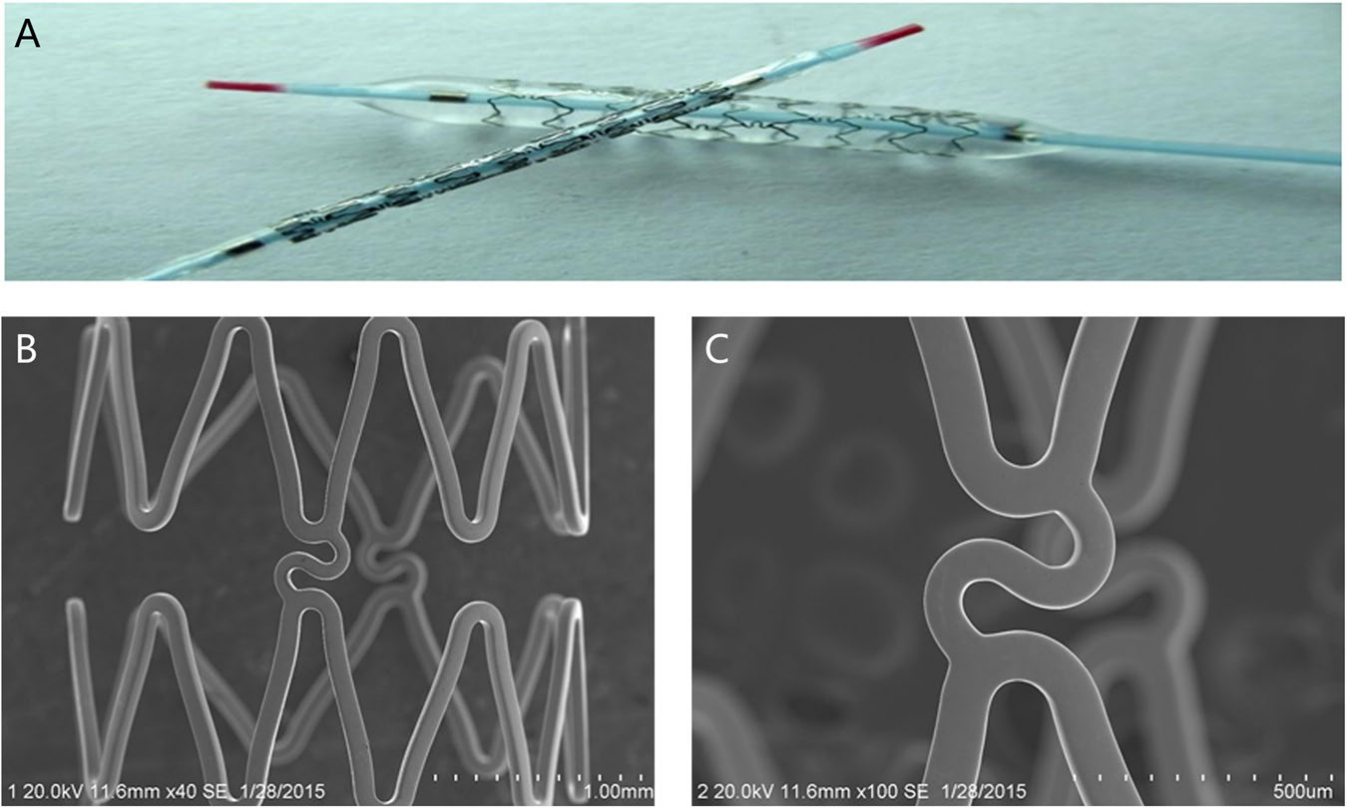
316L-Cu stainless steel bare metal stent: (**A**) General photo; (**B**) SEM photo; (**C**) SEM photo of strut at higher magnification.

In order to investigate whether 316L-Cu have the function of re-endothelialization, 316L-Cu-BMS were successfully fabricated by our group (Fig. 8). 316L-Cu was processed into tube and then laser cut to fabricate stent. Commercial available 316L-BMS (SINO Medical Sciences and Technology INC, China) and Firebird 2 DES (Micro Port, China) were used as control. The size of all the stents was 3.0 mm × 15 mm.

### Animals

The animal study was approved by the Institutional Animal Care and Use Committee of Peking University First Hospital (Code J201559) and conformed to the Guide for the Care and Use of Laboratory Animals in Beijing. Eight Chinese Miniature Swine (30 kg to 55 kg and six months to eight months) were purchased from China Agricultural University, China. The gender of the animals was ignored and they were fed a standard laboratory chow diet without lipid.

### Stent implantation

There are total of eight 316L-BMS, eight Firebird 2 DES and eight 316L-Cu-BMS. Three types of stents were randomly assigned and placed in the left anterior descending (LAD), circumflex (LCX), or right coronary artery (RCA) in one animal. In order to decrease the incidence of acute thrombosis, premedication with 300 mg aspirin (Bayer, Germany) and 75 mg clopidogrel (Xin Li Tai Pharmaceutical Co. Ltd, China) was administered before stent implantation for one day. Aspirin (100 mg/d) and clopidogrel (75 mg/d) were then given until sacrificed. Subcutaneous injection of 0.3 mg/kg ketamine (Double-Crane Pharmaceutical Company, China) was administered to all animals prior operation for basic anesthesia, and then intravenous injection of 0.05 g/L sodium pentobarbital (Peking University First Hospital Animal Center, China) was given to keep the status of anesthesia and to extend the experimental time. Onwards, the groin of the pig was fixed and sterilized, and the femoral artery was punctured with Seldinger method. Then, 6F sheath was inserted with intravenous injection of heparin (100 U/kg). With 0.035 inch guide wire, 6F JL 3.5 and JR 3.5 guiding catheters were used for coronary angiography. According to the target vessel diameter, the stent size was selected (stent expanded diameter: vascular diameter, 1.1:1–1.2:1). Guide wire (0.014 inch) was sent to the distal coronary artery. Along the guide wire, the stent was pushed to the target vessel site and released by using 8 atm–12 atm single expansion for 5 s–10 s. The angiography was repeated to make sure that no serious dissection and thrombosis occurred. Then the sheath tube was withdrawn, and the puncture site was compressed to stop bleeding for 20 min-30 min, with intramuscular injection of 8 × 10^4^ U/kg penicillin for preventing infection.

### QCA evaluation

At 14 day of implantation, operation for repeat coronary angiography on 8 animals was performed. QCA was performed by CAAS 5.9 QCA Software (PIE MEDICAL IMAGINE, Netherlands). Coronary artery measurements including baseline vessel diameter, DR, I-MLD, RVD, R-MLD and LL were recorded.

### OCT evaluation

At 14 day of implantation, OCT on 8 animals was conducted. OCT was performed by image analysis workstation (C7-XR Dragonfly) and the data including MSA, MLA, MinSA, MinLA, MinLD, MNA, and AS.

### SEM observation

SEM was performed to analyze endothelial coverage and morphology of endothelial cell layer. Intact stented vessels for microscopic observation were longitudinally bisected to expose the lumen surface and photographed. Specimens were rinsed in 0.1 mmoL/L sodium phosphate buffer (pH 7.2 ± 0.1) and then post-fixed in 1% osmium tetroxide for about 30 min. They were then dehydrated in a graded series (50%, 60%, 70%, 80%, 90%, and 100%) of ethanol. After they had been dried at the critical point, the tissue samples were mounted, sputter-coated with gold and observed under an SEM (Hitachi S-3000N, Japan).

### Histopathological evaluation

At 14 day of implantation, all the animals were sacrificed one after another. The heart was removed and perfused with heparin saline for 5 min at the pressure of 100 mmHg (1 mmHg = 0.0133 kPa). The stent vessel segment was separated rapidly and fixed with 10% formaldehyde solution. The samples with stent were embedded by methyl methacrylate (Prepared by Peking University School of Stomatology), and sliced (50 μm) with a hard tissue microtome (LEICA, Germany). All sections were stained with HE, and specimens were prepared for light microscopy (LEICA, Germany) examination. Stent endothelialization score was defined as the extent of the circumference of the arterial lumen covered by the endothelial cells and graded from 1 to 3 (1 = 0–25%, 2 = 25–75%, 3 = 75–100%).

### Statistical analysis

Values in normal distribution were expressed as mean ± standard deviation (SD). Independent-sample T test was used to analyze the statistical difference *in vitro*. Group imaging and integral data *in vivo* were analyzed with one-way ANOVA test. n representing the number of stents of different types. The value of P < 0.05 was considered statistically significant. All statistical analysis were performed by commercially available SPSS 14.0 system software (IBM, USA).

## Electronic supplementary material

Supplementary Fig. S1
