# Supplementary material for: Evaluation of promoting effect of a novel Cu-bearing metal stent on endothelialization process from in vitro and in vivo studies
Source: Sci Rep. 2017 Dec 12;7:17394. doi: 10.1038/s41598-017-17737-9 (PMC5727161; doi:10.1038/s41598-017-17737-9)
Supplement: Supplementary file 1 — Supplementary Fig. S1 [file 41598_2017_17737_MOESM1_ESM.docx]

# Supplementary Information

**Evaluation of promoting effect of a novel Cu-bearing metal stent on endothelialization process from *in vitro* and *in vivo* studies**

Shujing Jin^1,2,+^, Xun Qi^3,4,+^, Bin Zhang^5,+^, Ziqing Sun^1^, Bingchun Zhang^1^, Hui Yang^1^, Tongmin Wang^2^, Bo Zheng^5^, Xingang Wang^5^, Qiuping Shi^5^, Ming Chen^5,^*, Ling Ren^1,^*, Ke Yang^1^ & Hongshan Zhong^3,4,^*

^1^Institute of Metal Research, Chinese Academy of Sciences, 72 Wenhua Road, Shenyang 110016, China

^2^School of Materials Science and Engineering, Dalian University of Technology, Dalian 116024, China

^3^Department of Radiology, The First Affiliated Hospital of China Medical University, Shenyang 110001, China

^4^Key Laboratory of Diagnostic Imaging and Interventional Radiology of Liaoning Province, The First Affiliated Hospital of China Medical University, Shenyang 110001, China

^5^Peking University First Hospital, Department of Cardiology, Beijing 100034, China

* Corresponding authors.

E-mail addresses: cm6141@sina.com (M. Chen), lren@imr.ac.cn (L. Ren) and hszhong@cmu.edu.cn (H. Zhong)

^+^ Shujing Jin, Xun Qi and Bin Zhang equally contributed to this work.


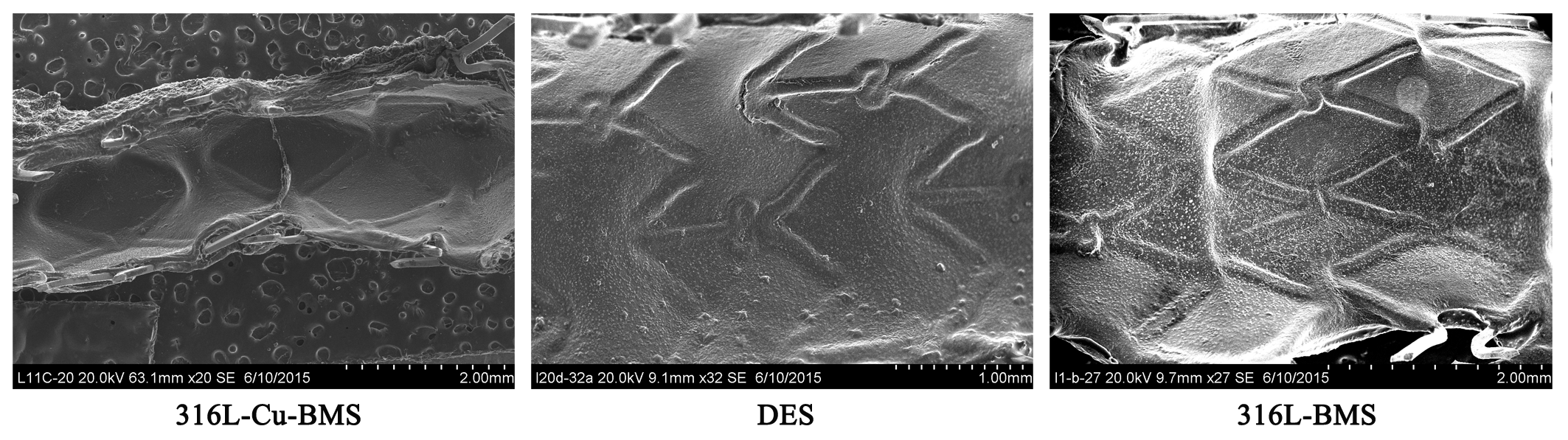


**Supplementary Fig. S1.** SEM images of different stents in animal artery after 14 days intervention.
